# A Multi-Agent Gamification System for Managing Smart Homes

**DOI:** 10.3390/s19051249

**Published:** 2019-03-12

**Authors:** Alicja Winnicka, Karolina Kęsik, Dawid Połap, Marcin Woźniak, Zbigniew Marszałek

**Affiliations:** Institute of Mathematics, Silesian University of Technology, Kaszubska 23, 44-100 Gliwice, Poland; Alicja.Lidia.Winnicka@gmail.com (A.W.); Karola.Ksk@gmail.com (K.K.); Marcin.Wozniak@polsl.pl (M.W.); Zbigniew.Marszalek@polsl.pl (Z.M.)

**Keywords:** heuristic, gamification, Internet of things, artificial intelligence, multi–agents solution

## Abstract

Rapid development and conducted experiments in the field of the introduction the fifth generation of the mobile network standard allow for the flourishing of the Internet of Things. This is one of the most important reasons to design and test systems that can be implemented to increase the quality of our lives. In this paper, we propose a system model for managing tasks in smart homes using multi-agent solutions. The proposed solution organizes work and distributes tasks to individual family members. An additional advantage is the introduction of gamification, not only between household members, but also between families. The solution was tested to simulate the entire solution as well as the individual components that make up the system. The proposal is described with regard to the possibility of implementing smart homes in future projects.

## 1. Introduction

Increasingly, intelligent technologies are present in our lives. Examples are telephones, televisions and other household appliances. However, along with the upcoming 5G (5th Generation) mobile network standard, large hopes are related to the Internet of Things [1]. The Internet of Things is nothing but the Internet where the recipients and senders of information are connected devices. Examples of such devices are the above-mentioned smartphones. Each device acquires data from the environment using built-in sensors, such as cameras, microphones, and motion and temperature sensors. Information often has to be processed to extract data, which are used by other objects in the network. This solution allows improving our life [2].

Smart homes are just one such example, where systems installed in the home are able to monitor the entire area in the absence of owners, or analyze and modify existing conditions. The conditions are understood as temperature analysis, water heating, or food control in a refrigerator. It all increases not only the comfort of life, but also minimizes energy consumption or the amount of food thrown away [3]. The Internet of Things can significantly change our lives, but to make this possible, there is a need to create systems that will monitor and supervise all devices. Moreover, the exchange of information by network users may be insufficient. If a current or future failure is detected, the user of the entire system should be able to receive such information. Moreover, such information should be transmitted not only within the installed technology, but anywhere. Hence, the need to design different interfaces and present data to the user using a smartphone or laptop so that he can intervene in different situations.

The vision of a home that helps users in performing tasks may be interesting, not only by controlling tasks, but also by motivating them to perform. Motivating can be solved by introducing the technique of gamification, that is, broadcasting additional content in order to gain some experience or points. This type of solutions may prove to be practical if gamification is subject to a certain group of users. In this paper, we propose a multi-agent system that is responsible for controlling the performance of simple tasks at home through installed sensors. In addition, the system introduces the aforementioned competition between the household members. In addition, children are taught duties at home and observe them.

From a technical point of view, the proposed solution is a multi-agent system for the purpose of exchanging information obtained by agents. This is important because the flat or house in which the system is installed can be very large, and tasks can be performed in different places. The advantage of the proposed idea is the use of popular equipment such as smartphones to obtain information because of their popularity.

The article is divided into several sections to increase readability. In Section 1, we introduce to the topic. In Section 2, we present the structure of the proposed system. Section 3 is devoted to the allocation of tasks for users, and then the agent’s operation based on artificial intelligence methods (in particular artificial neural networks) is described. The next sections present the conducted experiments and their discussion.

## 2. Related Works

Intelligent technologies are strongly developed in the world of science. Above all, aspects are related to increasing the quality of our lives. The most developed branches of such systems are energy and security fields. In both cases, the proposed architectures are different in terms of not only the operation but also used technology. In the case of intelligent energy management systems, scientists focus on optimizing different parameters to reduce consumption, which in turn reduces production costs [3]. The second most popular aspect of the Internet of Things are the security features. A simple alarm informs us about a possible break-in, but does not analyze other situations. This is particularly evident in homes with a large area, the coverage of which can be expensive. For this purpose, different communication architectures between devices are created, or even information is exchanged so different cases can be analyzed. Intelligent software having access to cameras can process the image and register unplanned guests and notify owners. In terms of security, first, methods of artificial intelligence are used, which can quickly classify data. In particular, it is worth paying attention to the fact that the recording from the camera is a set of frames [4]. In terms of security, digital attacks are also an important issue, as smart homes are protected and monitored in a digital way [5]. Researchers analyze and build complex systems that can operate on data not only on given devices (edge technologies), but using external devices such as cloud or servers [6]. Scientific research is carried out not only in terms of safety, but also environmental protection and health of residents. An example is air pollution system, where contaminants can get inside by opening a window or door [7].

These systems present different approaches to implementation and construction. However, similar ideas are used in the topic analyzed in this article, i.e., a system based on a certain criterion of gamification. In [8], the authors described the idea of using gamification for rule management. First, the authors focused on the integration of user interfaces with appropriate motivating techniques. This is important due to the age of users, where it will be a big determinant not only to design the interface, but also to choose a strategy to motivate users to act. In contrast, the authors of [9] showed the technique of constructing rules in such systems to involve all participants of the house to cooperate. Again, in [10], the risks associated with the installation of such systems are described. Particularly, the aspects of motivation are important to not discourage action, which may result from assigning too difficult tasks to specific household members.

In this paper, a different approach is presented by using sensors in popular devices and their common communication by placing a single point with a knowledge database and using artificial intelligence methods. In addition, we present the method of assigning tasks to individual household members, taking into account various factors such as age, type of performed work or commitment.

## 3. Proposed Gamification System

The main idea is to create a home management system for the smallest tasks such as picking up garbage, to repairing items that have broken or are close to such a state. Imagine a situation that a client buys a smart home for his family. The system should supervise the situations in the home. In the case of some inaccuracies, it should inform the household members as well as make sure that everything functions optimally. Unfortunately, some tasks can be avoided by family members or be put off to be done later. A trivial example is watering flowers.

For the system to function, all tasks should be in the database from which they are broadcast, depending on the time of execution. The basic database of tasks should be available to the client, which he can be modified as a system administrator. Each task is composed of several elements, of which the basic are the name, frequency of repetition, occurrence (in which days) and the prize. The administrator can modify the frequency and repeatability, but only to a certain extent. Some tasks such as watering flowers should not occur too often, therefore any deviations from the norm should be accepted as higher, that is, above the level of one house. This is similar for the prize: within the house itself, prizes can be allocated in any way, but not if in competition, as houses on the whole street could be competing.

It is easy to see that the competition takes place on two levels: between members of a single house, as well as among different houses. This type of competition would motivate family members in all homes, especially if, after a certain time, the results are evaluated and the highest sum of points highlighted in a certain way.

The reward for completing the task is the number of points, which are calculated on the basis of several factors such as difficulty level ϵ, the minimum and maximum number of points to win θ, execution time *t* and the priority ζ. Some of these conditions depend on the status of specific family members, for example, some task would be more difficult to do for a child than for an adult, thus its level of difficulty would increase when the child is a user. This would result in a higher reward that can be obtained for this task. Such a way of evaluating and rewarding tasks would help encourage children to develop through performing more and more difficult and demanding home duties and other tasks included in the database.

The level of difficulty is not the only parameter on which the reward depends, but the only one whose value is determined by the user’s status. The other parameters are independent of the person performing the task. They can be determined by persons supervising the system (for example, parents) or directly by a program that would determine a specific value on the basis of other coefficients.

One of independent parameters of the user’s status is the maximum and minimum number of points per task. Both values are set in advance and they are in the range θ∈〈1,10〉. The maximum amount allows preventing too fast progress, thus increasing its safety. For example, points scored too quickly by a child could result in choosing too difficult tasks for him that he could not cope with physically or mentally (e.g., he could be harmed while trying to do it). To prevent this, we set a minimum number of points. It is a protection of the program that sets up the prize under certain conditions. The simplest and most general situation is to assign a task to a user who has already performed this task many times. In this case, the level of difficulty is very low, which results in an adequately low reward. Too few points could discourage the user from completing the task (and, in the case of home duties, it would not be good because it still needs to be done). The minimum number determines the smallest number of points that a user can get for completing this task—no matter how many times he has already done it and how simple it is.

Priority is another important parameter to determine the award, which is specified in the range of ζ∈〈0,max〉. The total value is independent of the difficulty and constant for each task. It depends only on the day or time when the task has to be done—for example, certain tasks are more important on Saturday, and not on Tuesday, thus on Saturday they have the value max, and on Tuesday 0.5·max. For those with higher priority, there is a correspondingly higher prize, which should encourage the user to do it as soon as possible at the most appropriate time.

The time is another parameter participating in the description of the task. It is predetermined for each task and prevents the user from increasing the tendency to procrastinate. It does not directly affect the reward, but rather is its opposite. It can result in punishing the user with negative points (which affect not only his own score, but also the score of the whole house). For not completing the task within a given time, the user receives a warning (depending on the preferences) or loses points, and, as a last resort, a notification is sent to the person supervising the system. If the task is completed at a certain time, the obtained parameters are compared to the properties of the task. Depending on their fulfillment, reward points are assigned. This parameter is dependent on parameters such as the time interval—some tasks should be performed in exact hours on a specific day. An example is making dinner, which should be done in the afternoon when the family members return from work.

Obviously, some tasks should appear to be done automatically when appropriate factors occur. Laundry is an example. Depending on the amount of dirty clothes, e.g., if it exceed a certain weight, the task “laundry” should be created.

This way of assigning tasks allows earning reward points by individual members of the house, which are added to the account of the whole house. The gamification system allows various simple tasks and prevents them from being omitted. Moreover, it introduces into the life of the members additional elements such as competition or motivation.

## 4. A Multi-Agent Idea for Assigning Tasks

We assume that the described gamification has the right to exist at the system, which will try to search for the task itself. Obviously, those tasks should make sense. That is why we propose a system based on the operation of multi-agents. Formally, an agent is a system that is placed in a given environment. In addition, it may be autonomous, communicate with other agents, and achieve specific goals.

A multi-agent system is a system based on a set of agents who cooperate with each other to increase efficiency or achieve goals [11,12,13,14,15]. In our case, the system must supervise assigning tasks and exchange data between agents at home. Moreover, the additional task is to send the data from a given house to make possible competition between whole houses in a particular area.

Imagine the house in which most of the tasks are analyzed automatically. To make this possible, agents will be located in many places. It indicates that they will be responsible for the specific area. Each agent saves data about the task execution by any user, thus there is no one agent assigned to a specific user. However, the system being installed will not contain as many agents as users, but a much larger number of agents. The reason for this is the ability to control other equipment or rooms. This number depends on the functionality of the system.

To illustrate such a situation, let us assume that the system works in a house inhabited by one person. Assume that one of the basic tasks is watering flowers. The system cannot acknowledge the task to be finished if the user waters flowers only in one room, because there are sensors. A similar situation occurs when the flowers are in several rooms and the system calculates points for watering each flower. These types of things should be handled by the operation of multi-agents, where each of them will collect data and process it according to current needs.

**Figure 1:**
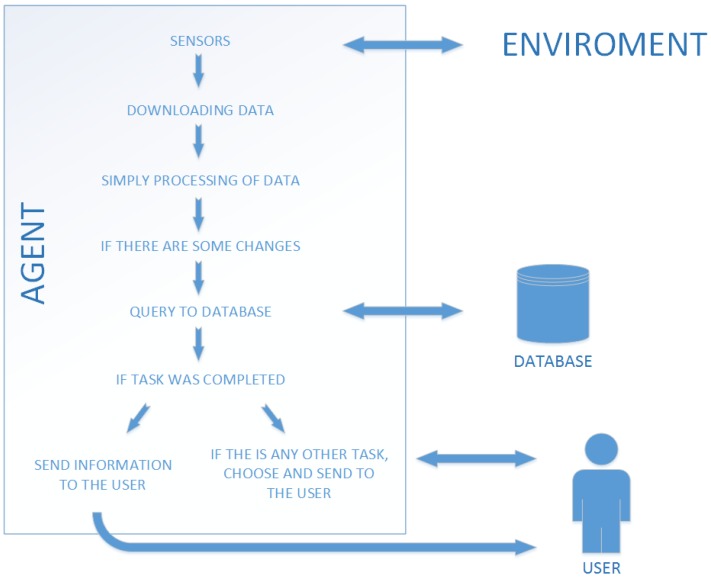
Agent architecture in the proposed system.

The proposed solution is that we will treat an agent as a single instance of the program, which sends and gets the information from the database. The agent has the task to retrieve data using built-in sensors, and then to process them. The processed information is compared to the previously obtained data (the interval between downloading may depend on a specific time interval or on the occurrence of movement—for this purpose, additional sensors are required). If there are any differences, the data are sent to the database. The database is built in the simplest relational model where many triggers are created. If the information in a given cell reaches the limit set in the task, the trigger returns information to the device that recently made the query. Received data may mean that the user has completed the task, and thus, an agent sends information to the user. If the task is completed, the database sends information about the remaining ones. The agent generates a task based on the received information from the database. The illustration of these actions is presented in Figure 1.

**Figure 2:**
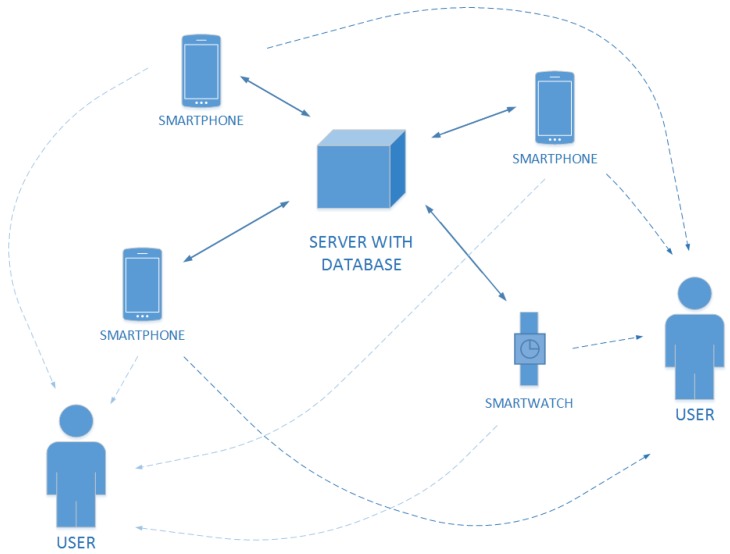
Visualization of communication between components in the proposed system.

This allows creating many agents whose communication will be indirect through an external, common information database (see Figure 2). Each agent can download a list of current tasks and modify it. In addition, each agent should control the current status of the apartment with the help of built-in sensors. If the agent has access to motion sensors, in the case of any movement, it should be analyzed and the results should be saved in the database. If the data are updating, each agent is informed about it (through triggers). Each update also checks if the task is completed. In the case, when there is a change of data and the task is performed, such information is saved in the database and information about it is returned to the user. Obviously, the prizes are given.

### 4.1. Task Handling

In the described idea, we have several agents who register and process data and send them to the database. Communication takes place through the central point, which is the database. The great advantage of such an action is to relieve agents of additional information as well prevent their duplication throughout in the system. The description of the operation and construction of agents is presented in the following sections, where we focus on the idea of gamification and the mechanics of tasks operation.

#### 4.1.1. Generating New Tasks

Generating tasks consists in entering new tasks manually by the user, or by introducing a new agent into the system. The first case is a simple operation, where the user enters the task with all data into the system, and it can be accepted or rejected by the other houses. If we create a task only for one house, no reward points will be assigned to it. The situation can be changed if there is no competition with other houses—it is possible when there are no other system users (interpreted as houses, not household members), or when there is not any shared tasks.

The second case is to introduce a new agent into the system. It is also very simple—while adding new agents, there is requirement to give information about the database, where the data should be sent. Depending on the installed functionality, the completed tasks will be built into the agent. Connection to the database is defined as sending its own tasks to database—if they already exist, the data on the agent is updated. Otherwise, the new tasks is inserted into the database.

#### 4.1.2. Assigning Tasks

The information about users is used to assign tasks. The same task can be assigned to several people. When multiple people do one task, the reward is shared among all of them.

Every day, a list is created with all tasks that have to be done this day. Whether the task is carried out on a working day or on a day off, it will have different difficulty level ω. The basic distribution of this value may be as follows

(1) $$\omega =\left\{\begin{array}{cc} \mathrm{manual}\;\mathrm{labour} & \omega =0.8\\ \mathrm{intellectual}\;\mathrm{work} & \omega =1\\ \mathrm{school} & \omega =1.2 \end{array}\right.$$

The above values have been chosen in an empirical way to differentiate the effort of individual people. Furthermore, the age coefficient ξ of every user is taken into account. If ξ is greater than the required age limit, it is set to a constant value, for example ξ=10. Additionally, the equation takes into account the health status μ of the user in the range 〈0,1〉, which, respectively, means sick and healthy. Another parameter is the current point status ϕ of the user, which is initially equal to 1 (the value is not zero to prevent dividing by zero). These data are used to calculate the current state of the user by the following equation

(2) $$\Xi =\frac{\sqrt{\xi }\cdot \xi \cdot \mu }{\omega \cdot \phi }.$$

It is easy to notice that the state of every user depends on the amount of earned points, which is the reason for relieving the most distinguished members of the household.

**Figure 3:**
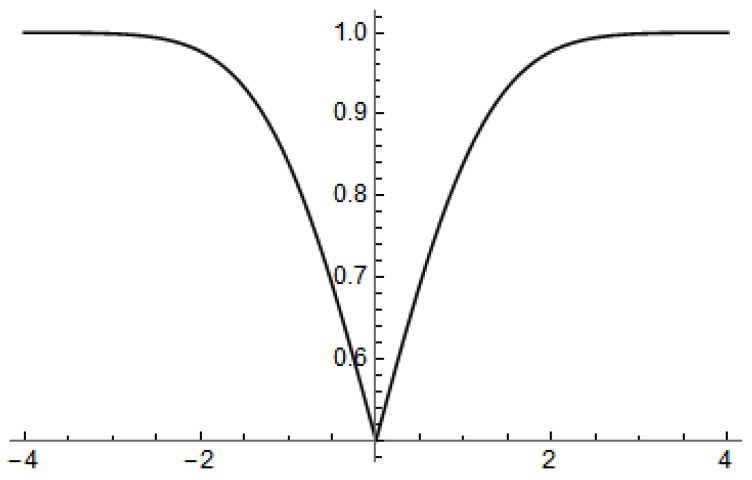
Chart of function Q(·) for assigning tasks, where $\frac{1}{n}\sum_{i=1}^{n}\left|1-Q\left(x_{i}\right)\right|=0.5$.

In the next step, the stack *R* with potential rewards is created and it is used in calculation the value of function Q(·) for each undone task *k* in database in the following way

(3) $$x_{k}=\gamma_{1}\cdot \frac{R[k]}{10}+\gamma_{2}\cdot \frac{\max_{i\neq k}R\left[i\right]}{\Xi },$$

where $\gamma_{1}$ and $\gamma_{2}$ are the percentage contribution of components, where these values fulfill the equation $\gamma_{1}+\gamma_{2}=1$. The obtained value is used in calculating the value of the function described as

(4) $$Q\left(x_{k}\right)=\int_{x_{k}}^{\infty }\frac{1}{2\pi }\exp\left(\frac{-x_{k}^{2}}{2}\right)dx_{k}\approx \left[\frac{1}{\left(1-a\right)\cdot x_{k}+a\cdot \sqrt{x_{k}^{2}+b}}\right]\frac{1}{\sqrt{2\pi }}\exp\left(\frac{-x_{k}^{2}}{2}\right),$$

where *a* and *b* are coefficients, mostly $\frac{1}{\pi }$ and 2π. Using these values, the best task is chosen according to

(5) $$\max_{k}\eta_{k}\cdot Q_{n}\left(x_{k}\right)$$

where $\eta_{k}\in \langle 0,5\rangle$ is the priority assigned to *k*th task and a function $Q_{n}\left(x_{k}\right)$ [16] reflects the chart (see Figure 3) to prioritize tasks with small prizes but a high priority. It can be described as

(6) $$Q_{n}\left(x_{k}\right)=\left\{\begin{array}{c} \left|1-Q(x_{k})\right|\;\;\;\;\mathrm{if}\;\left|1-Q(x_{k})\right|\geq \frac{1}{n}\sum_{i=1}^{n}\left|1-Q\left(x_{i}\right)\right|\\ \left|Q(x_{k})\right|\;\;\;\;\;\mathrm{if}\;\left|1-Q(x_{k})\right|<\frac{1}{n}\sum_{i=1}^{n}\left|1-Q\left(x_{i}\right)\right| \end{array}\right..$$

The use of the function Q(·) is for normalization of values, and then to allow assigning tasks at any priority.

## 5. The Operation of Agent

The object, which will be placed in the house and will have sensors for analyzing the environment, will be called an agent. The data will be recorded depending on the equipment of the agent. Among the most popular and most commonly used agents, we can distinguish smartphones, cameras, motion sensors, gas sensors, etc. The choice of how the data are processed depends on the type of sensors. The most frequently used data are images (camera recording) and numerical data (weight and temperature). To speed up analysis of various data, we propose the use of machine learning, in particular artificial neural networks. In the proposed system, each agent retrieves information, processes it and communicates with the database. This solution means that the implemented software can be the same. In addition, the agent’s operation and the used method will only depend on the sensors that are build-in.

The idea is based on the assumption that each agent could process the obtained data by using a neural network and the results will be placed in the database. If the task criteria are fulfilled, the task status in the database is changed to executed. Then, the reward is assigned to the appropriate person and that person is sent a notification. However, in this model, we do not consider any punishments for not carrying out the task. That is the reason for our proposition to check all records in database every few hours.

### 5.1. Machine Learning Approach

**Figure 4:**
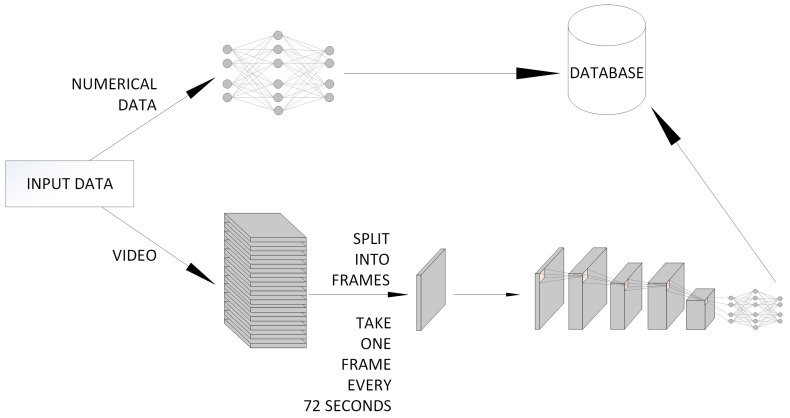
Graphical representation of the division of samples into classifiers.

Unfortunately, there is no single universal method for the processing various types of data. Therefore, we propose a hierarchical structure consisting of two types of neural networks, where one of them will process graphics, and the other numerical values. The diagram of this idea is presented in Figure 4. However, there is no way to create one classifier that will process everything. Thus, we propose a following solution: one classifier for one particular problem. In fact, such an idea is possible because each agent will take care of specific tasks. For many tasks, the calculations are performed and records in the database are modified depending on the results.

#### 5.1.1. Neural Network for Numerical Data

Artificial neural networks are inspired by the operations of the neurons in human brain. The idea is to create a grid of smaller objects connected to each other for data processing [17]. Formally, the network is a set of columns (called layers) that are created by smaller elements, which are neurons. There are three types of layers: input, hidden and output. The first one is responsible for accepting input values, hidden layers process values, and the output one returns the result. Neurons between the layers are connected with each other by synapses, which are modeled as a connection with an assigned weight, i.e., a random value in the range 〈0,1〉. Therefore, the neuron is the smallest element of the network that receives data, processes it and returns the result of its calculations. The values of neurons from the previous layer and the set of weights assigned to the connections between them may be called as input data for next layer. Let us denote the output from the *i*th neuron as $z_{i}$, and the weight as $w_{i}$, then the value of the neuron will be calculated as the sum of the products of these values normalized by the sigmoidal function f(·) as follows

(7) $$f\left(\sum_{i=0}^{n-1}w_{i}\cdot x_{i}\right)=\tanh\left(\sum_{i=0}^{n-1}w_{i}\cdot x_{i}\right),$$

where *n* is the number of neurons in the previous layer.

The most commonly used training algorithm is backpropagation. This process is about modifying the weights on connections in the whole network with respect to the loss function, defined as half of the square of the difference between expected value from the network and obtained one

(8) $$E=\frac{1}{2}\sum_{i=0}^{k-1}{\left(y_{i}^{expected}-y_{i}^{actual}\right)}^{2}.$$

Depending on the given modified weights in each layers, the above equation can be interpreted differently. The modification of weights between *j*th neuron in the output layer and *k*th neuron in the hidden one is performed as follows:

(9) $$\Delta w_{kj}=-\alpha \cdot \frac{\partial E}{\partial w_{kj}},$$

where α is a learning rate. To derive this equation, we use the rule chain, which is defined as

(10) $$\frac{\partial E}{\partial w_{kj}}=\frac{\partial E}{\partial y_{j}}\cdot \frac{\partial y_{j}}{\partial x_{j}}\cdot \frac{\partial x_{j}}{\partial w_{kj}}.$$

Thus, using the definition of a neuron and simple transformations, we obtain the following formula

(11) $$\frac{\partial E}{\partial w_{kj}}=-\left(t_{j}-y_{j}\right)\cdot y_{j}\cdot \left(1-y_{j}\right)\cdot y_{k},$$

where $y_{j}$ is the value of *j*th neuron.

If the neuron *j* is in the hidden layer, the calculation of weight will be as follows

(12) $$\frac{\partial E}{\partial w_{kj}}=-\sum_{i\in I_{j}}\left(\delta_{i}w_{ji}\right)\cdot y_{j}\cdot \left(1-y_{j}\right)\cdot y_{k},$$

where $\delta_{i}$ is error value of *i*th layer.

#### 5.1.2. Neural Network for Graphical Data

The image is perceived by the computer in a different way than the human eye. A two-dimensional image for a computer is a tensor, an object containing three components needed to describe it, including height, width and depth. As depth, we mean the individual layers of the image. For example, an image considered in terms of the RGB model will have three components, where each corresponds to one of the colors. The objects described in this way are used in the classification of graphics by convolutional networks. They are mathematical structures inspired by the functioning of the visual cortex [18].

The mathematical model includes the construction of three layers. The first of these is the convolutional layer, which works as an image filter. The layer has one task, i.e. to process all layers of the image. Processing is based on the displacements of the filter described as a matrix of the size k×k. In fact, a single image covered with a filter matrices can be interpreted as a one large grid of rectangles. Each of these rectangles is assigned a weight *w*, which is a numeric value used in the training process described later. Furthermore, this part of image can be interpreted as a neuron, which value is calculated as

(13) $$f\left(\sum_{i=0}^{k}\sum_{j=0}^{k}w_{i,j}\cdot a_{x+i,y+j}\right),$$

where $a_{x+i,y+j}$ is the value returned by the neuron at position (x,y).

The second type of layers is the layer called pooling, which is designed to reduce the size of images by feature extraction. A given matrix of the size m×m is moved over the image, where, depending on the chosen function, one of the values in this matrix is transferred to the new image. The mentioned function can be a maximum condition that selects the pixel with the highest value on the matrix. The third type of layers is called fully-connected, which is the classic neural network described in the previous subsection. Constructing such a network often requires the use of the dropout technique, i.e., removing neurons which value is lower than the threshold. In practice that means simplifying the neural network for removing non-essential elements.

The convolutional neural network returns the value assigned to a given class. If the network is designed to classify 10 objects, there will be 10 neurons on the output layer and the classes will be labeled with a vector consisting of single one and nine zeros. To normalize the output from the network, a function called softmax is used.

Training such a construction is possible by using various algorithms that modify the weights (which were given in random way) between neurons. It is an optimization task, where weights are modified to minimize the loss function (often the difference between the expected value and the received value). One of this algorithm is RMSProp [19]. An algorithm is based on calculating two statistical parameters, which are variation *v* and mean *m* in each iteration *t* as

(14) $$m_{t}=\beta_{1}\cdot m_{t-1}+\left(1-\beta_{1}\right)\cdot \nabla f\left(w_{t-1}\right),$$

(15) $$v_{t}=\beta_{2}\cdot v_{t-1}+\left(1-\beta_{2}\right)\cdot {\left(\nabla f(w_{t-1})\right)}^{2},$$

where $\beta_{1}$ and $\beta_{2}$ are coefficients. These two values are used in the process of updating weights $w_{t}$ as

(16) $$w_{t}=w_{t-1}-\frac{\lambda }{\sqrt{v_{t}+\epsilon }}⊙m_{t},$$

where λ is a training rate and ϵ is a small constant value (usually equal to $10^{-6}$) that prevents division by 0.

## 6. Experiments

To evaluate the proposed method, two groups of tests were carried out. The first consisted in creating agents and testing various configurations of the proposed techniques due to accuracy, as well as the sense of practical use. The individual components were implemented in JAVA, C# languages, Mathematica 11 and the classic mysql database was used. After preparing the exemplary functionality, a simple system was implemented with available devices such as smartphones and motion sensors. The tests were carried out in three different apartments.

### 6.1. Evaluation of the Proposed Method

In the first step, the basic information that can be taken by the agents were selected and three tasks were added:

- light meter—control of light in the room; and
- video—watering plants, taking out the trash.

**Figure 5:**
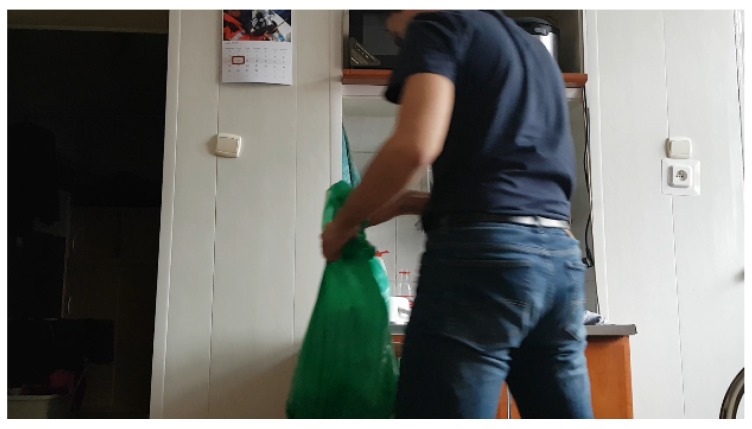
An exemplary frame used in the training process to detect the execution of a task.

**Figure 6:**
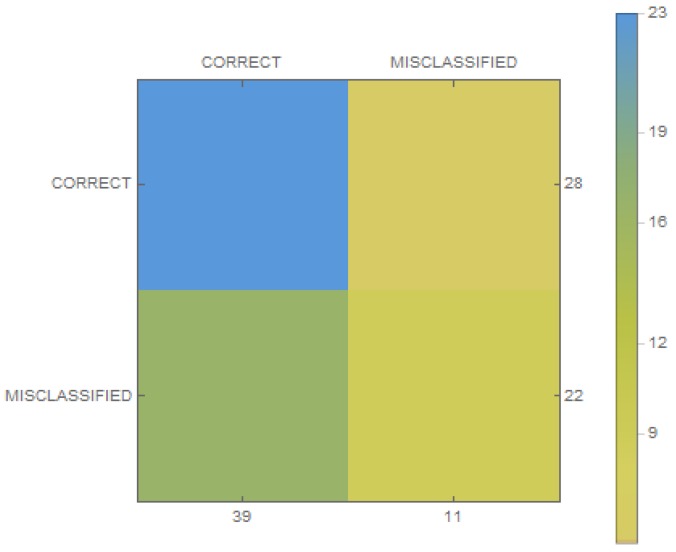
Confusion matrices for classic neural network.

Neural networks were used to classify these three tasks, due to the large amount of computing power required. The classic neural network was used to detect data on the light meter. The built-in sensor was aimed at the bulb in the room. The obtained data were recorded over a period of time, for example 3 s. The data were saved to a numerical vector. In addition, two items were added: time of day (morning, 0; noon, 0.4; evening, 0.7; and night, 1), which was stamped on the current time, and the presence of people in the room. The presence was checked using a motion sensor that records infrared radiation, which was returned as a numeric value 0 (meaning no movement) or 1 (meaning movement) every 3 s. These three values were saved in the vector that was used in the training process. The time of day is an important element when sensors are in the bedrooms. We assumed that the task of saving light was sent if there was no movement in the room and the light was on. We established 50 such samples, 35 of which were used in the training process in the proportions 80:20 (training samples to verification). The classifier structure was composed of four layers, where two of them were hidden and contained four neurons. Then, it was trained to get an error of 0.01. After that, effectiveness was checked for the entire database (an example is presented in Figure 5), and the results are shown in Figure 6. The resulting efficiency for such a small database was exactly 78%, which is not a high score, although it should be noted that there were few training data. The exact results of statistical measurements are shown in Table 1. It is worth noting that the false omission rate was over 27%, which indicates the proportion of false negatives that are falsely rejected.

**Figure 7:**
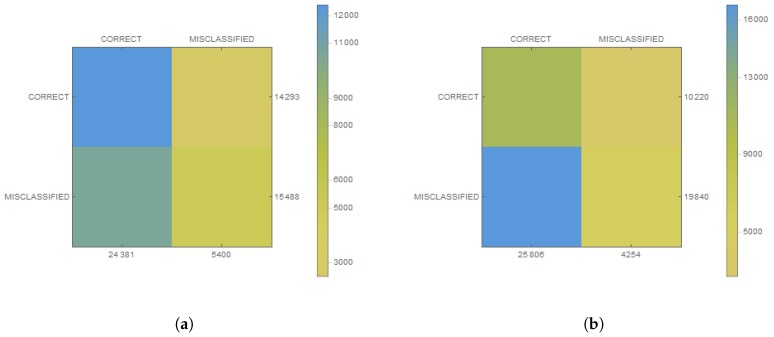
Confusion matrices for convolutional neural network: (**a**) watering flowers; and (**b**) taking out the trash.

The analysis of the behavior of people, e.g., taking out trash or watering flowers, was much more complicated. For this purpose, there was recorded a database of 150 video files (the size of 148×148) with people taking out trash. Each video lasts on average 10 s. Each of the videos was divided into 24 frames, which were used in the process of training the convolutional network. If the person was on the image with a garbage bag, the photo was labeled as trash. Similarly, training data for flower watering were prepared—the person had to hold a bottle of water. In both cases, a network was used with a similar structure, as is shown in Table 2. The networks were trained to achieve an effectiveness of more than 90% in the 80:20 ratio (training samples to verification) with only 130 video files. The obtained training charts are shown in Figure 7. Then, the database was extended by additional 20 unused video files in the training process. Then, real effectiveness was checked along with statistical measurements in Table 1. For both networks, the achieved efficiency was over 80% and the precision was above 86%. It is worth noting that the probability of rejecting a false sample was very large.

### 6.2. Case Study

To check the operation and impact of the system, three families including children (aged 15–18 years) were recruited for a 48 h test and to express their opinions.

#### 6.2.1. Main Goals

To check the real performance of the proposed system using available equipment, the following questions were asked

- Is the system of assigning tasks working correctly? Are the tasks assigned to children within their abilities?
- Is the proposed gamification solution useful? Does it result in better performance of basic tasks at home?
- Does the system work correctly? How does the placement of agents affect its operation?

#### 6.2.2. Preparation of the Environment

**Figure 8:**
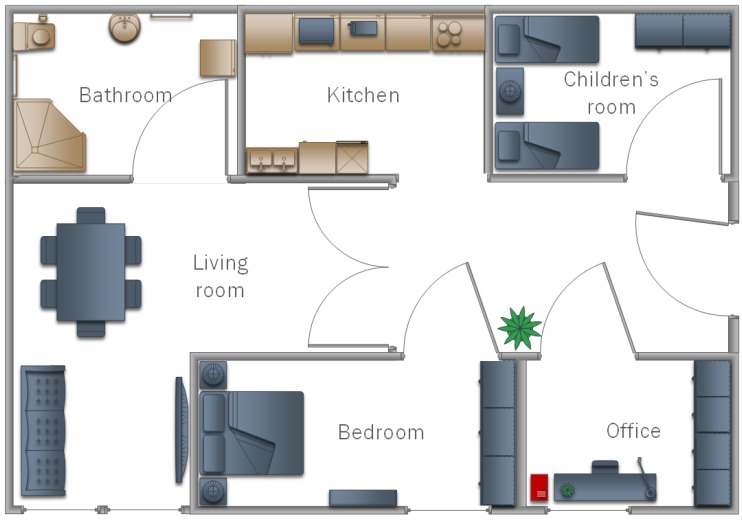
A model of the home that was used in testing proposed model in Experiment I.

**Figure 9:**
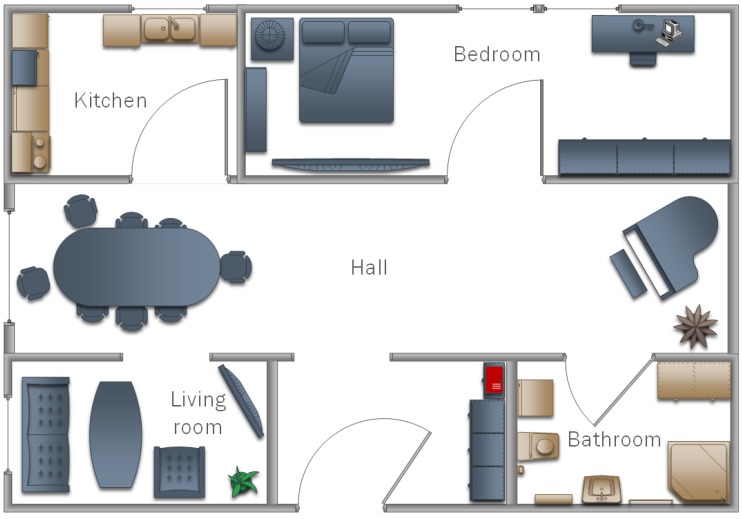
A model of the home that was used in testing proposed model in Experiment II.

**Figure 10:**
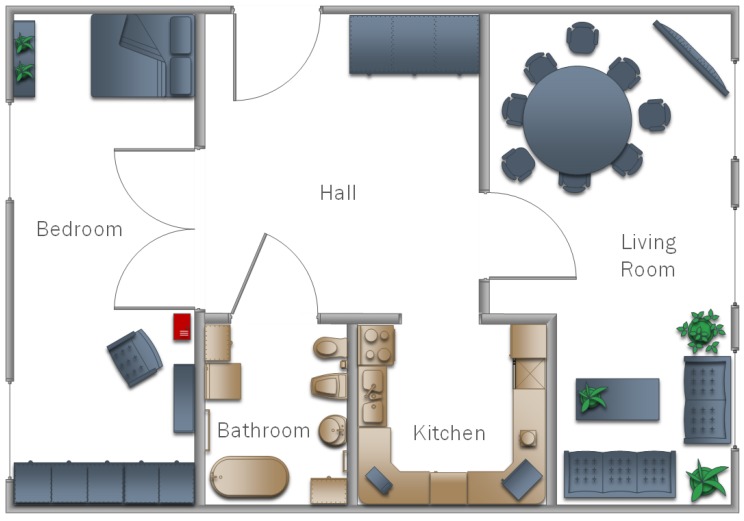
A model of the home that was used in testing proposed model in Experiment III.

The classifiers prepared in this way were used to check the operation of the multi-agent system in practice. Three different flats were used in the research, the structures of which are shown in Figure 8, Figure 9 and Figure 10. In each of the flats, a server was set up where the database was maintained. Additionally, in the kitchens and salons, there aws a smartphone hung on the wall to record video data relative to watering flowers, as well as taking out the trash. In addition, in the room for children and bedrooms, smartphones were suspended and set to lamps as well as motion sensors. Each device was treated as an agent, thus the access to the network and the application in the background were set. They processed the obtained data and sent them to the database. Examples of tasks were sent in the form of a text message to smartphones of the household.

#### 6.2.3. System Evaluation

Three families that agreed to take part in software testing were asked to complete the questionnaire, which is presented along with the average results in Table 3.

Based on the obtained results, we can answer the questions described in Section 6.2.1. The system of assigning tasks was assessed very well, which was confirmed by the results of questionnaire as well as opinions. It was pointed out that the tasks were repeated at regular intervals, or if one was done. There was not a situation that there were two tasks. The parents pointed out that the children were primarily given the task of watering flowers, and the parents took out the rubbish. It was well received, because it was noted that smaller children should not receive such a task. The second highly valued element was the gamification, which resulted in a lot of fun during the competition for earning points and as a result of such action the lack of problems with garbage was pointed out. Unfortunately, it was also noticed that children wishing to get extra points watered flowers even without the task. This note indicates that users are eager to earn points, thus a richer task database is advisable.

The functioning system was the worst rated element. Based on the results and statements of the testers, the system does not always correctly classify the performance of tasks by using the image. The reason may be a bad sensor setting or location. In the case of agents working during light control, users were satisfied. However, they pointed out that at night the system does not work optimally: the light turned on late at night was not controlled by agents.

## 7. Conclusions

Intelligent technologies enter our lives faster and faster by integrating multi-agent systems into various types of equipment. A much larger growth is planned along with the arrival of the 5G network, which will increase development opportunities through a higher speed and a much larger number of connected objects to the network in a small area. In this paper, we show that the multi-agent system can be used to oversee the performance of simple household duties. An especially important aspect was the gamification scheme, which mobilized the family members to overcome them in time. This solution allowed automatic assignment of tasks depending on many parameters. This was important due to the age of the household—for the youngest, tasks will be simpler. Simpler does not mean less demanding, because the tasks should enable the child to develop. In addition, we showed that the operation of many agents is a valuable solution in the application of a large area, where different agents could analyze the same task (thanks to the use of an external database). The proposed solution was tested by simple elements, but nothing stands in the way of expanding the entire model by adding more functionality. 

## Figures and Tables

**Table 1:** Statistical parameters for all tested classifier.

	NN for Controling the Light	CNN for Watering Flowers	CNN for Taking out the Trash
Accuracy	0.78	0.818676	0.858483
Sensitivity	0.793103	0.779742	0.746325
Specificity	0.761905	0.863244	0.93415
Precision	0.821429	0.867138	0.884344
Negative predictive value	0.727273	0.773954	0.845161
Miss rate	0.206897	0.220258	0.253675
Fall-out	0.238095	0.136756	0.06585
False discovery rate	0.178571	0.132862	0.115656
False omission rate	0.272727	0.226046	0.154839
F1 score	0.807018	0.821121	0.809494

**Table 2:** Structure of convolutional neural network used for image classification.

Layer	Output Shape
Convolutional	(None,148,148,32)
Activation	(None,148,148,32)
MaxPooling	(None,74,74,32)
Convolutional	(None,72,72,32)
Activation	(None,72,72,32)
MaxPooling	(None,36,36,32)
Convolutional	(None,34,34,64)
Activation	(None,34,34,64)
MaxPooling	(None,17,17,64)
Flatten	(None,18496)
Dense	(None,64)
Activation	(None,64)
Dropout	(None,64)
Dense	(None,1)
Activation	(None,1)

**Table 3:** Question and answers questionnaire on a scale of 〈0,10〉.

Question	House I	House II	House III	Average
System operation in terms of waste disposal	4	3.4	6	4.47
Operation of the system in terms of watering flowers	6	6	7	6.33
System operation in terms of light control	8.6	8	7.4	8
Automatic assignment of tasks	8	9	8	8.33
Points calculation	7	6	6	6.33
Motivation thanks to competition	9	8	8	8.33
